# Are fear memories erasable?–reconsolidation of learned fear with fear-relevant and fear-irrelevant stimuli

**DOI:** 10.3389/fnbeh.2012.00080

**Published:** 2012-11-19

**Authors:** Armita Golkar, Martin Bellander, Andreas Olsson, Arne Öhman

**Affiliations:** Department of Clinical Neuroscience, Karolinska InstitutetStockholm, Sweden

**Keywords:** reconsolidation, extinction, fear learning, fear-relevant, SCR, FPS

## Abstract

Recent advances in the field of fear learning have demonstrated that a single reminder exposure prior to extinction training can prevent the return of extinguished fear by disrupting the process of reconsolidation. These findings have however proven hard to replicate in humans. Given the significant implications of preventing the return of fear, the purpose of the present study was to further study the putative effects of disrupting reconsolidation. In two experiments, we assessed whether extinction training initiated within the reconsolidation time window could abolish the return of fear using fear-relevant (Experiment 1) or fear-irrelevant (Experiment 2) conditioned stimuli (CS). In both experiments, participants went through conditioning, extinction, and reinstatement testing on three consecutive days, with one of two reinforced CS being reactivated 10 min prior to extinction. We found that a single reminder exposure prior to extinction training did not prevent the return of extinguished fear responding using either fear-relevant or fear-irrelevant CSs. Our findings point to the need to further study the specific parameters that enable disruption of reconsolidation.

## Introduction

Pavlovian fear conditioning involves learning the relationships between aversive events (unconditioned stimuli, US) and the environmental stimuli (conditioned stimuli, CS) that predict such events. Although such learning represents an adaptive process that promotes survival, persistent conditioned responding in the absence of a CS-US contingency can develop into pathological anxiety. In fact, conditioned fear is regarded as one of the primary mechanisms in the aetiology of fear-related anxiety disorders (Mineka and Zinbarg, 2006) and Pavlovian fear conditioning represents a leading paradigm to study the neural mechanisms through which such fears are acquired and stored. Recent advances within the field of human fear learning suggest that acquired fear memories can be erased by interfering with the process of reconsolidation (Kindt et al., 2009; Schiller et al., 2010), during which acquired fear memories are temporarily modifiable (Nader, 2003; Dudai, 2004; Alberini, 2005). The main objective of the present study was to investigate the boundaries of disrupting reconsolidation.

In clinical practice, fear-related anxiety disorders are effectively treated by cognitive behavioral therapy (Barlow, 2002), which derives its effectiveness from the repeated exposure to the feared object in the absence of aversive outcomes. The experimental analog of exposure therapy is represented by the process of fear extinction in which the expression of a conditioned response (CR) is weakened through repeated exposures to the CS in the absence of the aversive US. According to an influential account proposed by Bouton (2002), extinction represents an inhibitory learning process involving learning of a new “safety memory” that competes with the original CS-US learning. Thus, according to this view, extinction does not cause an erasure of the original memory trace, but promotes learning of a new, CS—no US association (Bouton, 2002). This inhibitory learning theory of extinction is supported mainly by the recovery of the previously extinguished CR. This recovery can be attained in three principal ways; *spontaneous recovery* that develops with the passage of time (Rescorla, 2004) *reinstatement* following re-exposure to the US in the absence of the CS (Rescorla and Heth, 1975), and *renewal* by change of context (Bouton and Bolles, 1979).

Although the recovery of conditioned fear responding after extinction points to the persistence of learned fears, it does not necessarily imply that fear memories are irreversible. In fact, previous research using Pavlovian fear conditioning protocols in rats has shown that upon retrieval, consolidated fear memories can return to a labile state in which they are susceptible to disruption (Nader, 2003; Dudai, 2004; Alberini, 2005). In this process of reconsolidation, an already consolidated memory, after being reactivated by a reminder cue, transiently returns to a labile state, and requires new protein synthesis to persist. This is supported by findings in rats showing that intra-amygdala infusions of a protein synthesis inhibitor (anisomycin) immediately, but not 6 h, after reactivation of the fear memory significantly reduced conditioned fear responses at a later retention test (Nader et al., 2000). Subsequently, Ledoux, and colleges showed that both systemic and intra-amygdala injection of the β-adrenergic receptor antagonist propranolol (Debiec and Ledoux, 2004) blocked reconsolidation, a finding that more recently was extended to and replicated in humans by Kindt et al. (2009) using systemic propranolol treatment.

A related line of research has demonstrated that replacing pharmacological treatment (i.e., propranolol) with extinction training yields similar results. Thus, extinction training initiated within, but not outside, the critical reconsolidation time window has been shown to attenuate or block the return of conditioned fear, as first described in rodents (Monfils et al., 2009) and later extended to humans using skin conductance responses (SCR) (Schiller et al., 2010) and amygdala activity (Ågren et al., 2012) as indices of fear. Importantly, the original study by Schiller et al. (2010) showed that the effect of extinction training initiated within the reconsolidation window persisted 1 year later as measured by a reinstatement test. In order to establish the specificity of these effects and to increase the clinical validity of their results, Schiller et al. (2010) investigated whether the observed fear blockade would be specific to one US predictive cue or if it would generalize to another associated cue, thus more resembling real-life situations in which fear can be elicited by multiple stimuli. To address this issue, they conducted a second experiment in which they used a within-subject design involving two differently colored squares (the CS + s) that were paired with an electric shock US, while a third square was never paired with shock (the CS−). Reactivation of one reinforced CS + (CS + r) and the CS—10 minutes prior to extinction on the second day of testing resulted in a significant reduction in skin conductance response to the CS + r compared to the non-reactivated CS + (CS + nr) in a subsequent reinstatement test. Thus, the authors concluded that extinction training disrupted reconsolidation and that this disruption specifically erased fear memory for the reactivated CS+. Similar findings were recently reported using the same within-subject design but replacing the electrical shock US with aversive sounds (Oyarzun et al., 2012). These authors reported that the CR on the first reinstatement trial was significantly reduced to the reactivated CS+ compared to the non-reactivated CS+.

If extinction training following the reactivation of a fear memory can not only facilitate new learning, but also cause an erasure of the fear memory, the clinical implications for the treatment of anxiety disorders could be profound. However, given that the feared object in clinical fears are more often fear-relevant than fear-irrelevant (Öhman and Mineka, 2001), the findings by Schiller et al. (2010) using colored squares as CSs should optimally be replicated using CSs that more closely resembles those that are the objects of clinical fears. Indeed, in an attempt to replicate and extend the experimental findings reported by Schiller et al. (2010), Kindt and Soeter conducted two separate studies (Kindt and Soeter, 2011; Soeter and Kindt, 2011) in which they tested whether reconsolidation of fear-relevant stimuli (pictures of spiders) could be disrupted by extinction training. The authors reported that extinction learning initiated within the reconsolidation window did not prevent the return of fear as measured by SCR, fear potentiated startle (FPS), or US expectancy ratings. As such, these results pose a challenge to the generalizability of the original findings reported by Schiller et al. (2010) and emphasize the need for replications. Given the significant implications of preventing the return of fear, the aim of the present study was to further study the putative effects of disrupting reconsolidation. More specifically, using the same within-subject design as reported by Schiller et al. (2010), we assessed whether extinction training initiated within the reconsolidation time window could abolish the return of fear using fear-relevant (Experiment 1) or fear-irrelevant (Experiment 2) CSs.

## Materials and methods

### Experiment 1

#### Participants

Nineteen participants (mean age *M* = 27.2, SD = 9.55; 9 men) were recruited through poster advertising on Karolinska Institutet campus. All participants gave written consent for their participation and were given three cinema tickets.

#### Stimulus material

Three different pictures depicting fearful male faces from the Karolinska Directed Emotional Faces (Lundqvist et al., 1998) served as CSs. For each picture, the background was removed and color was converted to grey-scale. Stimuli were presented in a pseudo randomized order with the criterion that there could be no more than two trials of the same CS in a row throughout the experiment. A white fixation cross was shown on a black background during the inter-trial intervals (ITIs), the duration of which varied between 10 and 18 s (*M* = 14) throughout all experimental sessions. The experiment was run in a sound-attenuated chamber on a desktop PC with a standard 21-inch cathode ray tube (CRT) monitor. Screen resolution was 800 × 600 pixels and the refresh rate was set to 60 Hz. The experiment was programmed in Presentation 13.1 (Neurobehavioral Systems, www.neurobs.com). Participants viewed pictures at a distance of about 1 m. The US was a 100 ms monopolar DC-pulse electric stimulation (STM200; Biopac Systems Inc., www.biopac.com) applied to the participant's right wrist. Startle probes were 50-ms bursts of 95-dB[A] white noise with a near instantaneous rise time (<1 ms). Startle probes were presented binaurally through headphones (Sennheiser HD202).

#### Measurements and recordings

The eye-blink component of the startle response was measured through electromyographic (EMG) recordings of the left orbicularis oculi muscle using two miniature Ag/AgCl electrodes prepared with electrolyte gel. A third ground electrode was placed behind the left ear over the mastoid. The raw EMG signal was amplified and filtered through a 28–500 Hz bandpass, which was rectified and integrated with a time constant of 20 ms. Startle eye-blink magnitude (microvolts) was measured as the amplitude from onset to peak and normalized using T-standardization resulting in a distribution with an overall mean of 50 and a standard deviation of 10 for each participant. Mean startle difference scores were calculated as [mean startle magnitude to startle probe in the presence of the CS] – [mean startle magnitude to startle probe during ITI for each session] as has been described previously (Norrholm et al., 2008). Each session started with the presentation of six noise-alone trials to allow for habituation to the startle sound. In each trial, the CS was presented for 6 s and the startle probe was presented 4–5 s following CS onset. Startle probes were presented on an equal number of trials of each CS and the ITIs (9 out of 12 trials during acquisition, 8 out of 12 during extinction, and 6 out of 9 during reinstatement testing).

SCR was assessed using two Ag/AgCl electrodes connected to the index and middle finger of the left hand. The SCR was measured for each CS trial as the base-to-peak amplitude to the first response (in microsiemens, mS) in the 0.5–4.5 s window after stimulus onset. The minimal response criterion was set to 0.02 ms and responses that did not pass this criterion were scored as zero. The raw SCR scores were square root transformed to normalize the distribution and range-corrected by dividing each participant's responses by their maximum US response.

#### Procedure

**Figure 1:**
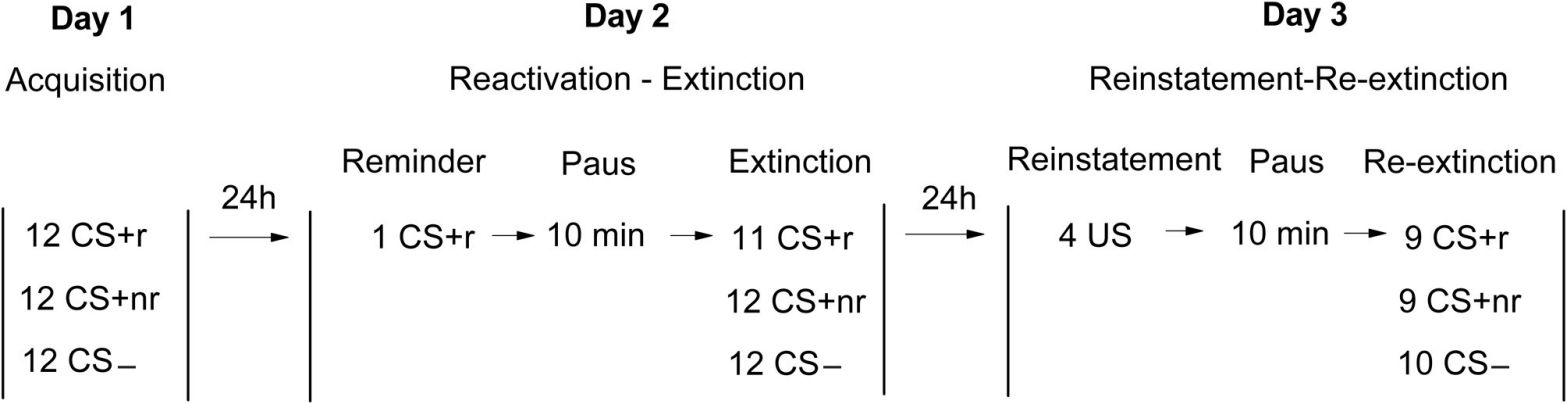
**Experimental design.** Acquisition, Extinction, and Reinstatement testing occurred on three consecutive days. On day 1, subject were fear conditioned to two different CSs (CS + r, CS + nr) that were followed by an electrical shock on 50% of the presentations whereas a control stimulus (CS−) was never followed by the shock (Acquisition). On day 2, subjects received one single reminder trial of the CS + r (Reactivation). This was followed by a 10 min pause after which subjects were exposed to repeated non-reinforced presentations of each CS (Extinction). On day 3, subjects received four unsignaled presentations of the US (Reinstatement). This was followed by a 10 min pause after which subjects again were exposed to repeated non-reinforced presentations of each CS (Re-Extinction).

The experiment was divided into three consecutive sessions conducted ~24 h apart: acquisition (Day 1); reactivation and extinction (Day 2) and reinstatement and re-extinction (Day 3,) using a within-subjects design (Figure 1) based on Experiment 2 in the study by Schiller et al. (2010). Briefly, subjects underwent fear conditioning using three different CSs. Two CSs (CS + r and CS + nr) were paired with the shock whereas the third CS (CS−) was never paired with the shock. A day later, subjects received a single presentation of CS + r but not the CS + nr. Ten min after the reminder trial, extinction training was conducted using repeated presentations of all CS without the aversive outcome. Finally, reinstatement of the fear memory was conducted 24 h later.

***Day 1: acquisition.*** On day 1 of the experiment, the intensity of the shock was adjusted individually for each participant by starting on a low level and gradually increasing the intensity up to a level that the participants experienced as “uncomfortable but not painful.” During acquisition, two different CSs (CS + s) co-terminated with a shock on 50% of each CS presentation and one CS was never coupled to a shock (CS−). Participants were informed that two of the faces *could* be associated with a shock, while one face would never be associated with shock. They were also instructed to pay attention to the contingency between the faces and the shock. In order to enhance the retention of the CS-US contingencies on the following day (Norrholm et al., 2006), at the end of acquisition, participants were instructed to remember what they had learned during the experiment.

***Day 2: reactivation and extinction training.*** In order to reactivate the acquisition memory, participants were exposed to a single unreinforced CS+ presentation (CS + r). Which CS+ that was reactivated was counterbalanced across participants. After the reminder presentation, participants were given a 10-min break during which they watched a pre-selected video clip (see Schiller et al., 2010) after which participants underwent extinction training consisting of non-reinforced presentations of all three CSs.

***Day 3: reinstatement and re-extinction.*** The reinstatement and re-extinction session began with four unsignaled presentations of the shock after which participants were given a 10-min break, again watching a video clip. Re-extinction followed immediately after the break and consisted of non-reinforced presentations of all CSs.

#### Statistical analyses

Data were analyzed with SPSS 17.0 for Windows. Each session of the experiment (acquisition, extinction, and reinstatement testing) was analyzed separately with repeated measures analysis of variance (ANOVA). Each phase of the experiment was blocked into an early and a late phase, calculated as the mean of the first three and last three trials respectively, following the practice of previous studies (Schiller et al., 2010). Consistent with the original study by Schiller et al. (2010), the first CS− trial was disregarded at the beginning of the reinstatement session. We adopted a significance level of 0.05 and report partial η^2^ as the estimate of effect size. Greenhouse-Geisser adjustments of degrees of freedom were used when appropriate. Significant interactions and pre-planned comparisons were followed up with separate two-tailed *t*-tests.

#### Results

***Startle.*** To assess fear acquisition and extinction, we measured startle differentiation between the two CS + s and the CS− in a Stimulus (CS + r, CS + nr, CS−) × Time (early, late) repeated measures ANOVA. During acquisition, FPS responses were overall significantly higher to the CS + s than to the CS− and these responses decreased from early to late acquisition, as supported by a significant main effect of Stimulus, *F*_(2, 36)_ = 10.32, *p* < 0.001, η^2^ = 0.36, and a significant main effect of Time *F*_(1, 18)_ = 14.52, *p* = 0.01, η^2^ = 0.45. To confirm that acquisition was successful for both CS + s, we compared the mean FPS to CS + r and CS + nr with the CS− during the late phase of the acquisition session. Subjects showed significantly stronger FPS responses to the CS + r than to CS−, *t*_(18)_ = 2.67, *p* = 0.02, as well as to CS + nr compared to CS−, *t*_(18)_ = 2.50, *p* = 0.02. Moreover, the level of acquisition to CS + r and CS + nr did not differ, *t*_(18)_ = 0.09, *p* = 0.40.

During extinction, there was a significant main effect of Stimulus *F*_(2, 36)_ = 5.19, *p* = 0.01, η^2^ = 0.22 and Time, *F*_(1, 18)_ = 88.30, *p* < 0.001, η^2^ = 0.83, and a marginally significant Stimulus × Time interaction, *F*_(2, 36)_ = 2.85, *p* = 0.07, η^2^ = 0.14, suggesting that the decrease in FPS differed between the CSs from early to late extinction. Importantly, we confirmed that extinction training was successful as there were no significant differences between the CS + r and the CS−, *t*_(18)_ = 1.35, *p* = 0.19 or the CS + nr and the CS−, *t*_(18)_ = 0.86, *p* = 0.40 during the late phase of extinction training. Also, the CS + r and the CS + nr did not differ significantly after extinction, *t*_(18)_ = 0.56, *p* = 0.58. Moreover, we confirmed that there was a significant decrease in FPS from late acquisition to late extinction to both the CS + r, *t*_(18)_ = 2.12, *p* = 0.05, and the CS + nr, *t*_(18)_ = 2.74, *p* = 0.01, but not to the CS− *t*_(18)_ = 0.17, *p* = 0.87.

**Figure 2:**
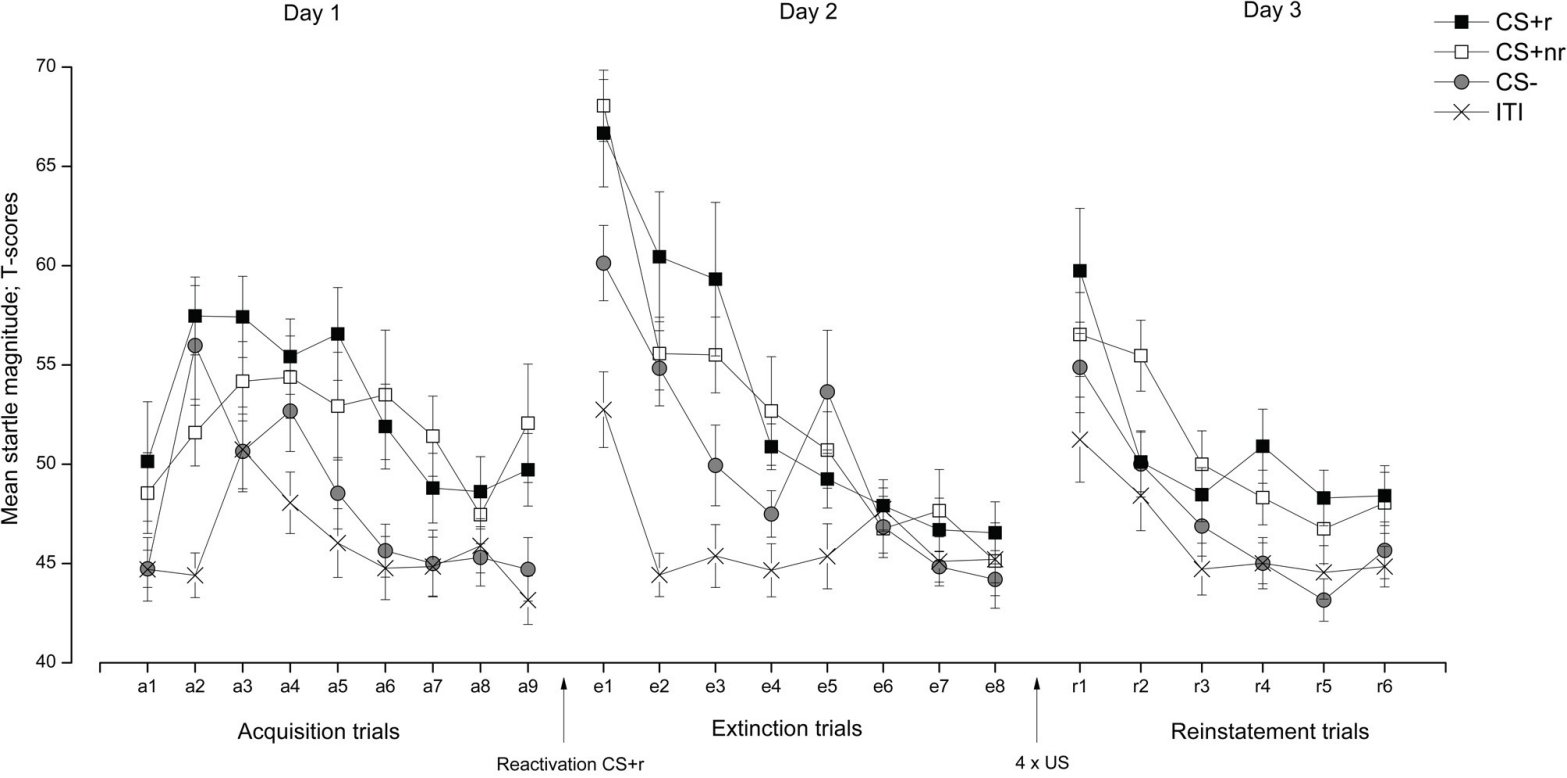
**Experiment 1: Reactivation of fear-relevant stimuli indexed by fear potentiated startle responses (FPS).** Mean startle response elicited during the presentation of the fear conditioned stimuli (CS + r, CS + nr), the control stimulus (CS−), and during the inter-trial intervals (ITI) during acquisition, extinction, and reinstatement testing. We did not observe any effects of fear memory reactivation (CS + r) prior to extinction learning on fear potentiated startle. Error bars represent standard error of the mean (SEM). Note that we did not present any startle probes during the reactivation trial.

Finally, the effect of the reactivation was assessed by comparing whether there was a reactivation-dependent change in FPS from the end of extinction (last trial) to reinstatement testing (first trial). This analysis revealed that all CS responses were overall larger during the first reinstatement trial than during the last extinction trial, as supported by a main effect of Time, *F*_(1, 17)_ = 22.11, *p* < 0.001, η^2^ = 0.57, in the absence of a main effect of Stimulus, *F*_(2, 34)_ = 1.61, *p* = n.s. Separate *t*-test showed that there was a significant increase in FPS from end of extinction to reinstatement testing for both CS + s (CS + r: *t*_(17)_ = 3.32, *p* = 0.01; CS + nr: *t*_(17)_ = 4.58, *p* < 0.001) and the CS− *t*_(17)_ = 3.74, *p* = 0.01, indicating a generalization of fear to the CS−. Therefore, we ran a second reinstatement analysis including only the CS + s (see Soeter and Kindt (2011) Experiment 2 for a similar approach). Re-analyzing reinstatement yielded a significant main effect of Trial, *F*_(1, 17)_ = 22.25, *p* < 0.001, η^2^ = 0.57, confirming that CR were overall higher during the first trial of the reinstatement test than during the last trial of extinction, and that there were no difference between the reactivated and the non-reactivated CS + [*F*_(1, 17)_ = 0.99, *p* = n.s.]. Thus, we did not observe a reactivation-dependent reinstatement of FPS, indicating a failure to interfere with the fear memory during the reconsolidation period. The FPS data are presented in Figure 2. For completeness, and in accordance with similar work (Schiller et al., 2010; Kindt and Soeter, 2011; Soeter and Kindt, 2011), we also ran the analysis after adopting selection criteria to establish that (1) conditioning was successful (defined as last two trials of acquisition > for both the CS + s vs. the CS−) and (2) extinction was successful (defined as the first two trials of extinction (startled trial 1–2) > the last two trials of extinction (startled trial 7–8). Adopting these selection criteria resulted in the exclusion of six subjects, but did not alter our reported findings. Analysis of the re-extinction data demonstrated a significant main effect of Stimulus *F*_(2, 34)_ = 5.35, *p* = 0.010, η^2^ = 0.24 and Time *F*_(1, 17)_ = 20.14, *p* < 0.001, η^2^ = 0.54, indicating that subjects discriminated between the CS + s and the CS− and that FPS responses decreased from early to late re-extinction training (see Figure 2).

**Figure 3:**
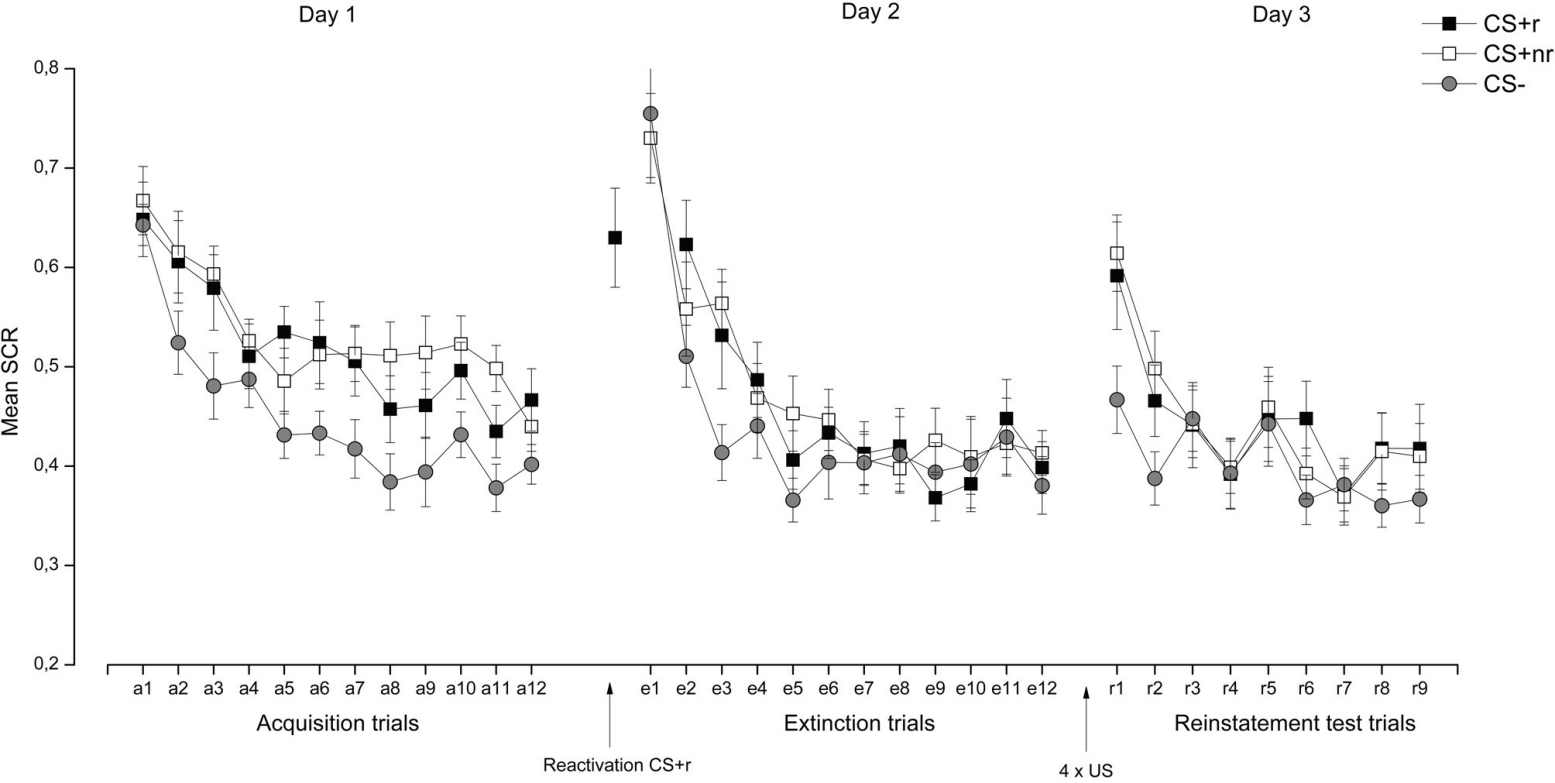
**Experiment 1: Reactivation of fear-relevant stimuli indexed by skin conductance responses (SCR).** Mean SCR during the presentation of the fear conditioned stimuli (CS + r, CS + nr), and the control stimulus (CS−) during acquisition, extinction, and reinstatement testing. We did not observe any effects of fear memory reactivation (CS + r) prior to extinction learning on SCR. Error bars represent standard error of the mean (SEM).

***SCR.*** Acquisition and extinction were analyzed in a Stimulus (CSr+, CSnr+, CS−) × Time (early, late) repeated measures ANOVA. During acquisition, SCRs were overall higher to the CS + s than to the CS− and these responses decreased from early to late acquisition, as supported by a significant main effects of Stimulus, *F*_(2, 34)_ = 12.85, *p* < 0.001, η^2^ = 0.43, and Time, *F*_(1, 17)_ = 51.63, *p* < 0.001, η^2^ = 0.75] (see Figure 3). Separate *t*-tests confirmed that there were significant differences between both the CS + r and CS−, *t*_(17)_ = 3.78, *p* = 0.002, and the CS + nr and the CS, *t*_(17)_ = 4.05, *p* = 0.001, during the late acquisition phase. The CS + r and the CS + nr did not differ significantly after acquisition [*t*_(17)_ = 0.94, *p* = 0.36].

During extinction there was a significant main effect of Time, *F*_(1, 17)_ = 48.16, *p* < 0.001. Follow-up *t*-tests indicated that extinction training was successful as there were no significant differences between the CS + r and the CS−, *t*_(17)_ = 0.30, *p* = 0.77 or the CSnr + and the CS−, *t*_(17)_ = 0.31, *p* = 0.76, during the late phase of extinction. The CSr+ and the CSnr+ did not differ significantly after extinction *t*_(17)_ = 0.01, *p* = 0.99. Moreover, we confirmed that there was a significant decrease in SCR from acquisition (late phase) to extinction (last trial) for both CS + r, *t*_(17)_ = 2.33, *p* = 0.03 and CS + nr, *t*_(17)_ = 2.37, *p* = 0.03, but no significant change in the CS− response, *t*_(17)_ = 0.99, *p* = 0.34.

Finally, the effect of reactivation was examined by assessing whether there was a stimulus-dependent change in SCR from end of extinction (last trial) to reinstatement testing (first trial), identical to previous reconsolidation studies (Schiller et al., 2010; Kindt and Soeter, 2011; Soeter and Kindt, 2011; Ågren et al., 2012; Oyarzun et al., 2012). This analysis revealed a significant main effects of Stimulus, *F*_(2, 34)_ = 5.48, *p* = 0.01, η^2^ = 0.24, and Trial, *F*_(1, 17)_ = 32.13, *p* < 0.001, η^2^ = 0.65 and a marginally significant Stimulus × Trial interaction *F*_(2, 34)_ = 2.94, *p* = 0.07, η^2^ = 0.15. Follow-up analysis showed that there was a significant increase in SCR to all three CSs [CS + r: *t*_(17)_ = 3.62, *p* = 0.01; CS + nr: *t*_(17)_ = 4.84, *p* < 0.001; CS−: *t*_(17)_ = 3.45, *p* = 0.01] but that the CR was significantly higher to both the reactivated *t*_(17)_ = 2.15, *p* < 0.05 and the non-reactivated CS + *t*_(17)_ = 3.80, *p* < 0.01 than to the CS− whereas there was no difference between the two CS + s [*t*_(17)_ = 0.47, *p* = 0.65]. Critically, even though the CS + r were reactivated, participants still showed a reinstated fear response to it, indicating a failure to interfere with the fear memory during the reconsolidation period. The SCR data are presented in Figure 3. For completeness, and in accordance with similar work (Schiller et al., 2010; Kindt and Soeter, 2011; Soeter and Kindt, 2011), we adopted selection criteria to establish that (1), conditioning was successful (defined as last two trials of acquisition > for both the CS + s vs. the CS−) and (2), extinction was successful (defined as the first two trials of extinction (trial 1–2) > the last two trials of extinction (trial 11–12). Adopting these selection criteria resulted in the exclusion of five subjects, but did not alter our reported findings. Analysis of the re-extinction data demonstrated a significant main effect of Stimulus *F*_(2, 32)_ = 9.38, *p* = 0.001, η^2^ = 0.37 and Time *F*_(1, 16)_ = 26.65, *p* < 0.001, η^2^ = 0.37, indicating that subjects discriminated between the CS + s and the CS− and that FPS responses decreased from early to late re-extinction training. Moreover, there was a marginally significant Stimulus × Time interaction *F*_(2, 32)_ = 2.53, *p* = 0.09, η^2^ = 0.14 driven by significant differentiation between the CS + s and the CS− during early [CS + r vs. CS−: *t*_(17)_ = 2.60, *p* = 0.02; CS + nr vs. CS−: *t*_(17)_ = 3.88, *p* = 0.001] but not late re-extinction training. There were no significant differences between the CS + r and the CS + nr during either early or late re-extinction.

### Experiment 2

In Experiment 2, we enrolled 20 new participants (mean age = 26.26, SD = 7.52; 9 males) and replaced the CSs used in Experiment 1 with fear-irrelevant CSs, i.e., colored squares. Also, to increase comparability with the original study by Schiller et al. (2010), and because it has been suggested that the presentation of startle probes may interfere with the measurement of SCR (Kindt and Soeter, 2011), we excluded startle probes in this second experiment and only measured SCR. All other experimental parameters and procedures were identical to Experiment 1.

#### Results

**Figure 4:**
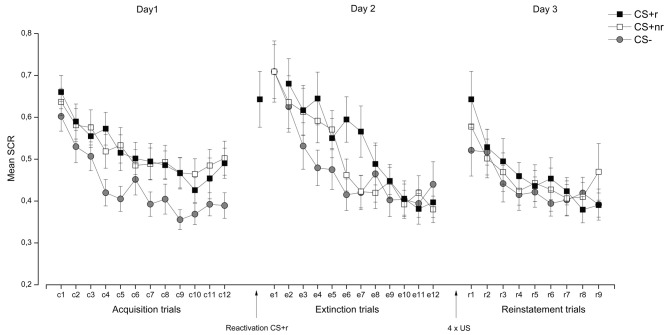
**Experiment 2: Reactivation of fear-irrelevant stimuli indexed by skin conductance responses (SCR).** Mean SCR during the presentation of the fear conditioned stimuli (CS + r, CS + nr), and the control stimulus (CS−) during acquisition, extinction, and reinstatement testing. Contrary to the findings by Schiller et al. (2010), we did not observe an effect of fear memory reactivation (CS + r) prior to extinction learning in SCR. Error bars represent standard error of the mean (SEM).

***SCR.*** Acquisition and extinction were analyzed in a Stimulus (CSr+, CSnr+, CS−) × Time (early, late) repeated measures ANOVA. During acquisition, SCR responses were overall higher to the CS + s than to the CS− and these responses decreased from early to late acquisition, as supported by significant main effects of Stimulus, *F*_(2, 38)_ = 12.61, *p* < 0.001, η^2^ = 0.40 and Time, *F*_(1, 19)_ = 37.64, *p* < 0.001, η^2^ = 0.67 (See Figure 4). Separate *t-tests* confirmed that there were significant differences between both the CS + r and CS−, *t*_(19)_ = 3.84, *p* = 0.001 and the CS + nr and the CS− *t*_(19)_ = 3.35, *p* = 0.01 during the late phase of the acquisition. The CS + r and the CS + nr did not differ significantly in the end of the acquisition phase *t*_(19)_ = 1.08, *p* = 0.29.

During extinction there was a significant main effect of Time, *F*_(1, 19)_ = 33.48, *p* < 0.001, η^2^ = 0.64]. Extinction training was successful as there were no significant differences between the CS + r and the CS−, *t*_(19)_ = 0.97, *p* = 0.36 or the CS + nr and the CS−, *t*_(19)_ = 0.90, *p* = 0.38, during the late phase of extinction. The CS + r and the CS + nr did not differ significantly after extinction *t*_(19)_ = 0.18, *p* = 0.86. Moreover, we confirmed that there was a significant decrease in SCR from acquisition (late phase) to extinction (last trial) for both CS + r, *t*_(19)_ = 2.74, *p* = 0.01 and CS + nr, *t*_(19)_ = 3.11, *p* = 0.01, but no significant change in the CS- response, *t*_(19)_ = 1.08, *p* = 0.29.

Finally, the effect of the reactivation was examined by assessing whether there was a stimulus-dependent change in SCR from end of extinction (last trial) to reinstatement testing (first trial). This analysis revealed a significant main effect of Time, *F*_(1, 19) = 9.13_, *p* = 0.01, η^2^ = 0.33, and a Stimulus × Time interaction, *F*_(2, 38)_ = 3.25, *p* = 0.05. Follow-up analysis showed that there was a significant increase in SCR to both CS + s [CS + r: *t*_(19)_ = 3.93, *p* = 0.01; CS + nr: *t*_(19)_ = 3.01, *p* = 0.01] but not to the CS− *t*_(19)_ = 0.96, *p* = 0.35 in the absence of significant difference between the two CS + s [*t*_(19)_ = 0.88, *p* = 0.39]. Thus, we did not find any support for the notion that reactivation of a conditioned fear memory can abolish the return of fear as measured by SCR. The SCR data are presented in Figure 4. For completeness, and in accordance with similar work (Schiller et al., 2010; Kindt and Soeter, 2011; Soeter and Kindt, 2011), we adopted selection criteria to establish that (1), conditioning was successful (defined as last two trials of acquisition > for both the CS + s vs. the CS−) and (2), extinction was successful (defined as the first two trials of extinction (trial 1–2) > the last two trials of extinction (trial 11–12). Adopting these selection criteria resulted in the exclusion of five subjects, but did not alter our reported findings. Analysis of the re-extinction data demonstrated a significant main effect of Stimulus *F*_(2, 38)_ = 3.28, *p* = 0.049, η^2^ = 0.15 and Time *F*_(1, 19)_ = 16.05, *p* = 0.001, η^2^ = 0.46 and a significant Stimulus × Time interaction *F*_(2, 38)_ = 4.20, *p* = 0.03, η^2^ = 0.18 driven by significant differentiation between the CS + s and the CS− during early [CS + r vs. CS−: *t*_(19)_ = 3.56, *p* = 0.002; CS + nr vs. CS-: *t*_(19)_ = 2.10, *p* = 0.049] but not late re-extinction training. There were no significant differences between the CS + r and the CS + nr during either early or late re-extinction.

## Discussion

Here we report that a single retrieval trial prior to extinction training did not disrupt the recovery of extinguished conditioned fear responses. These results stand in contrast to recent findings demonstrating that the expression of previously learned fears in humans can be blocked if extinction training is conducted during the reconsolidation time window (Schiller et al., 2010; Ågren et al., 2012; Oyarzun et al., 2012). These previous findings have received much attention, partly owing to their potential clinical implications (Pitman, 2011) but have proven hard to replicate (Kindt and Soeter, 2011; Soeter and Kindt, 2011). The inconsistencies between previous reports have been speculated to reflect procedural differences, such as the fear-relevant properties of the CSs and the use of concurrent indices of CR (Kindt and Soeter, 2011; Soeter and Kindt, 2011; Oyarzun et al., 2012). In the present study, we addressed these procedural differences in two separate experiments. We found significant return of fear to the reactivated and the non-reactivated CS + s using both fear-relevant (Experiment 1) and fear-irrelevant (Experiment 2) CSs. Thus, our results do not support the hypothesis that the failure to demonstrate that extinction training can disrupt reconsolidation is related to the fear-relevance of the CSs. Neither do our results support that the concurrent measurement of FPS, due to its intrinsically aversive nature, interferes with the measurement of SCR as we did not include auditory startle probes in Experiment 2 and still found significant reinstatement of SCR for both CS + s. A puzzling finding is however the generalization of CR to the CS− during reinstatement in Experiment 1. Thus, we observed a non-differential return of FPS to all CSs from extinction to reinstatement testing, whereas SCR responses were significantly higher to the two CS + s than to the CS−. Such generalization of CR was not observed during Experiment 2 in which we replaced the fear-relevant CSs with fear-irrelevant CSs. In fact, generalization of CR during reinstatement testing in humans has been reported previously (Milad et al., 2005; Dirikx et al., 2009; Soeter and Kindt, 2011) and may possibly involve the recruitment of non-associative process during acquisition or conditioning to the context given that the reinstatement USs are presented in the acquisition context. Importantly however, the reinstatement procedure used in the current study mimics that used in previous studies addressing reinstatement of human fears (e.g., Schiller et al., 2010; Golkar and Öhman, 2012; Oyarzun et al., 2012), which have demonstrated a specific return of fear to the CS+ compared to the CS−. This procedure has been validated by demonstrating the change in CR from end extinction to reinstatement testing is greater in the group receiving reinstatement USs than in a group not receiving unsignaled US presentations (e.g., Dirikx et al., 2004; Hermans et al., 2005; Norrholm et al., 2006). Moreover, for the interpretability of our data, the problem of a non-differential return of fear observed with FPS was not evident when CR was indexed by SCR in either Experiment 1 or in Experiment 2.

There are a number of experimental conditions, so called, *boundary conditions*, under which reconsolidation do not occur (Nader and Hardt, 2009). In the context of reconsolidation of fear memories in humans, the failures to replicate the disruption of reconsolidation by extinction training initiated during the reconsolidation time window have mainly been discussed in terms of two boundary conditions; acquisition memory strength, and memory updating mechanisms (see Kindt and Soeter, 2011; Soeter and Kindt, 2011; Oyarzun et al., 2012).

### Acquisition memory strength

It has previously been shown in rodents that stronger memories are more resistant to reconsolidation than weaker memories (Suzuki et al., 2004; Wang et al., 2009). For instance, auditory fear memories acquired with 10 CS-US pairings were shown to be stronger than with one single CS-US pairing, as indicated by less extinction following repeated CS-US pairings. These stronger fear memories, unlike the weaker memories, did not undergo reconsolidation (Wang et al., 2009). In the context of human reconsolidation studies, the strength of the acquired fear memory has been speculated to explain the failure to disrupt reconsolidation with fear-relevant CSs (Kindt and Soeter, 2011; Soeter and Kindt, 2011; Oyarzun et al., 2012). This assumption originates from work showing that fear-relevant CSs result in stronger fear memories than fear-irrelevant CSs, which is inferred from their resistance to extinction (Öhman and Mineka, 2001). A shortcoming of this line of reasoning is that in order to ensure successful acquisition and extinction, previous reported human reconsolidation studies (Schiller et al., 2010; Kindt and Soeter, 2011; Soeter and Kindt, 2011; Oyarzun et al., 2012; but see Ågren et al., 2012) have including only participants who showed successful extinction, i.e., that *did not* demonstrate resistance to extinction. Thus, it is unclear whether the argument that resistance to reconsolidation with fear-relevant CSs is mediated by the strength of the fear memory is relevant in the absence of an index of the acquisition memory strength (such as resistance to extinction). Obviously, a clarification of this issue requires a more direct assessment of the differences in associative strength obtained with fear-relevant and fear-irrelevant CSs. In sum, our results are not supportive of the conclusion that the fear-relevant properties of the CSs are sufficient to explain the difficulties in demonstrating that reconsolidation can be disrupted by correctly timed extinction training.

A related line of reasoning has focused on the between-studies differences in US reinforcement rate during acquisition of CR (Kindt and Soeter, 2011; Soeter and Kindt, 2011; Oyarzun et al., 2012). Whereas the studies by Schiller et al. (2010) and Oyarzun et al. (2012) used relatively low reinforcement rates during acquisition (37.5%), the studies by Kindt and colleagues (Kindt and Soeter, 2011; Soeter and Kindt, 2011) used 75% and 80% reinforcement rates respectively. The use of these higher reinforcement rates were recently suggested to have rendered the fear memories too strong and as such inhibited the induction of the reconsolidation process (Oyarzun et al., 2012). There are however at least three complicating issues to this interpretation. Firstly, it is not clear that higher reinforcement rates during acquisition actually produced “strong conditioning protocols,” as suggested by Oyarzun et al. (2012). As discussed above, there is no manifestation of strong conditioning (such as deficient extinction) or comparison between studies suggesting *stronger* conditioning obtained with the protocols used by Kindt and colleagues (Kindt and Soeter, 2011; Soeter and Kindt, 2011). Secondly, previous studies demonstrating the resistance to reconsolidation associated with stronger memories (Suzuki et al., 2004; Wang et al., 2009) were based on the number of CS-US pairing (i.e., acquisition was stronger with more CS-US pairings), rather than the proportion of reinforced trials. In this context it is noteworthy that the fewest number of CS-US pairings (i.e., four CS-US pairings) was reported by Kindt and Soeter (2011). Finally, contrary to the proposal by Oyarzun et al. (2012), previous research have shown that low reinforcement rates during acquisition results in slower extinction (Bouton, 2004). As such, the low reinforcement rate (37.5%) employed by Schiller et al. (2010) and Oyarzun et al. (2012) would be expected to yield stronger conditioning as indexed by more resistance to extinction than the reinforcement rates employed in the present study (50%) and in previous non-replications (75–80%; Kindt and Soeter, 2011; Soeter and Kindt, 2011). Perhaps most intriguing is the fact that a recent paper by Ågren et al. (2012), in which they successfully disrupted reconsolidation of fear memories, used a 100% reinforcement schedule with 16 reinforced CS+ presentations, ruling out that high reinforcement rates or many reinforced trials is sufficient to explain the failure to initiate reconsolidation.

### Memory updating

Memory updating has been suggested as a core boundary condition on reconsolidation (Lee, 2009) as engagement of reconsolidation mechanisms critically depends on whether a memory is being updated. For instance, it is more likely that memories undergo reconsolidation when the memory retrieval trial represents novel or relevant information (memory updating) as has been shown in both humans (Forcato et al., 2009, 2010) and non-human animals (Pedreira et al., 2004; see Lee, 2009 for a review). Moreover, the induction of reconsolidation requires that the reminder generates a mismatch between what is expected and what actually happens. Thus, CS offset, signaling the absence of the expected US and not CS onset, was required to trigger reconsolidation (Pedreira et al., 2004). In fact, it was recently shown in humans that retrieval *per se* is not sufficient to initiate reconsolidation (Sevenster et al., 2012) as updating of a fear memory did not occur under retrieval conditions in which the outcome was fully predictable. Following this line of reasoning, higher reinforcement rates during acquisition would be more likely to trigger reconsolidation given that a high CS-US probability during acquisition (100% CS-US contingency) would result in a greater mismatch at the non-reinforced retrieval trial (CS-no US). Thus, this stands in contrast to experimental results where studies with lower reinforcement rate are the ones showing successful disruption of the reconsolidation process, pointing to other factors being relevant. Apparently, the retrieval trial in our study was not sufficient to update the fear memory, but it is unclear exactly which experimental conditions that critically determined the failure to trigger reconsolidation in the present study. It is important to note that the failure to trigger reconsolidation in the current set of studies does not question whether disruption of reconsolidation can modify memories in humans in general, which has been demonstrated in several domains of memory research (see Schiller and Phelps, 2011 for a review). Neither do our data question that reconsolidation can be impaired in the more specific case of conditioned fear memories as reported by recent studies (Schiller et al., 2010; Ågren et al., 2012; Oyarzun et al., 2012). Our results do however raise the question of the extent to which the previously reported effects are stable enough to be translated into complex clinical settings, and address important questions regarding which boundary conditions that may explain the failure to reproduce the original findings by Schiller et al. (2010). It is also noteworthy that there are alternative approaches to interfere with reconsolidation of fear memories in humans, such as using propranolol administration in conjunction with memory reactivation (Kindt et al., 2009). This strategy was recently shown to effectively eliminate the return of FPS using the same experimental design as a reactivation-extinction procedure that did not eliminate the subsequent expression of fear (Soeter and Kindt, 2011). Given that propranolol was administered prior to memory activation, it has however been questioned whether these effects were mediated by effects of the drug on retrieval itself rather than by blocking of the reconsolidation of conditioned fear (See Schiller and Phelps, 2011 for a discussion).

## Conclusions/future directions

Taken together, the present study showed that a single retrieval trial prior to extinction training did not disrupt the recovery of extinguished conditioned fear responses to either fear-relevant or fear-irrelevant stimuli. Thus, replication failures like ours and those of Kindt and Soeter (Kindt and Soeter, 2011; Soeter and Kindt, 2011), alongside the strongly limiting boundary conditions of reconsolidation discussed here, raises the question whether the reconsolidation effects demonstrated by Schiller et al. (2010) are stable enough to be translated into the highly complex situations in which fears are acquired and expressed. This issue is further complicated by the fact that there are additional boundary conditions of reconsolidation including temporal parameters such as the age of the acquired memory; older memories have been suggested to be more resistant to undergo reconsolidation than more recently acquired memories (Suzuki et al., 2004; Frankland et al., 2006) (but see Debiec et al., 2002; Einarsson and Nader, 2012 for conflicting results), and retrieval trial duration; longer trial durations trigger extinction processes rather than reconsolidation (Pedreira and Maldonado, 2003). Moreover, although Schiller et al. (2010) showed that the memory reactivation effect lasted 1 year after acquisition, the effects reported by Oyarzun et al. (2012) were restricted to the first trial of reinstatement testing that occurred 48 h after acquisition. This discrepancy stresses the need for more research to evaluate the clinical potential of disrupting reconsolidation using extinction updating mechanism. Thus, in spite of considerable progress within the field of reconsolidation (Pitman, 2011) the translation gap to clinical applications remains immense. In fact, the critical differences between clinical disorders, such as post-traumatic stress disorder (PTSD), and laboratory experiments include many of the factors that have been defined as reconsolidation boundary conditions. For instance, compared to the laboratory experiments, PTSD often involves more complex CSs, stronger USs as well as a longer duration between memory formation and intervention and they are characterized by strong CRs and their resistance to extinction. Future research may help to advance our understanding of how the conditions for updating fear memory in humans can be optimized and ultimately help to bridge the gap between experimental research and the clinic.

### Conflict of interest statement

The authors declare that the research was conducted in the absence of any commercial or financial relationships that could be construed as a potential conflict of interest.
